# Editorial: New Insights Into Mechanisms of Epigenetic Modifiers in Plant Growth and Development

**DOI:** 10.3389/fpls.2019.01661

**Published:** 2020-01-10

**Authors:** Ming Luo, Gabino Ríos, Tomasz Jacek Sarnowski, Shoudong Zhang, Nitin Mantri, Jean-Benoit Charron, Marc Libault

**Affiliations:** ^1^ Key Laboratory of South China Agricultural Plant Molecular Analysis and Genetic Improvement, Guangdong Provincial Key Laboratory of Applied Botany, South China Botanical Garden, Chinese Academy of Sciences, Guangzhou, China; ^2^ Center of Economic Botany, Core Botanical Gardens, Chinese Academy of Sciences, Guangzhou, China; ^3^ Department of Citriculture and Plant Production, Instituto Valenciano de Investigaciones Agrarias (IVIA), Valencia, Spain; ^4^ Department of Protein Biosynthesis, Institute of Biochemistry and Biophysics Polish Academy of Sciences, Warsaw, Poland; ^5^ Centre for Soybean Research, State Key Laboratory of Agrobiotechnology and School of Life Sciences, The Chinese University of Hong Kong, Shatin, Hong Kong; ^6^ School of Science, The Pangenomics Group, RMIT University, Melbourne, VIC, Australia; ^7^ Department of Plant Science, McGill University, Sainte-Anne-de-Bellevue, QC, Canada; ^8^ Department of Agronomy and Horticulture, Center for Plant Science Innovation, University of Nebraska, Lincoln, NE, United States

**Keywords:** epigenetic regulation, plant development, histone modification, chromatin modification, DNA methylation, small RNAs

In eukaryotic cells, chromatin, a highly dynamic nucleoprotein complex, plays a critical role in controlling gene expression notably by regulating the interaction between transcription factors and regulatory elements. The structure of the chromatin is determined by epigenetic mechanisms, including DNA methylation, histone modifications, and chromatin remodeling. A growing body of evidence indicates that epigenetic regulations are involved in plant adaptation to environmental stresses, and in plant development, including flowering control, fruit and root development, as well as seed maturation and germination. Furthermore, epigenetic mechanisms have the potential to stabilize cell identity and maintain tissue organization. Hence, epigenetic diversity is now emerging as a new source of phenotypic variation to improve adaptation to changing environment and ensure yield and quality of crops. The 14 articles published in this Research Topic highlight recent progresses, opinions, and reviews to advance our knowledge in the role of the epigenome on controlling plant development, plant response to environmental stresses, and plant evolution. For instance, gene duplication and chromatin remodeling contribute to increase the morphological and cellular complexity of plants during their evolution according to Hajheidari et al.

Chromatin modifications, including DNA methylation and histone modifications, are critical in regulating gene transcription, and thus may reprogram cell differentiation and development (Inácio et al.; Zhang et al.; Hajheidari et al.). For instance, Inácio et al. immunolocalized various epigenetic marks and correlated epigenomic changes with transcriptional regulation when studying cork formation and quality in cork oak, a genuinely forest-specific process. Furthermore, changes in the acetylation levels of the lysine 9 of the histone H3 (H3K9) and lysine 5 of the histone H4 (H4K5) were found associated with the heat stress-dependent inhibition of lateral root formation in maize (Zhang et al.). Interestingly, whereas a global increase in histone acetylation was observed in response to heat stress, H3K9 and H4K5 acetylation decreased significantly in the promoter region of the haem oxygenase-1 (*ZmHO-1*) and giberellic acid–stimulated like-1 (*ZmGSL-1*) genes, two inhibitors of lateral root formation (Zhang et al.).

Plant cells have the capability to dedifferentiate in totipotent cells, a prerequisite to asexual embryogenesis. Recent papers support a role of histone deacetylation and DNA methylation in cellular reprogramming leading to callus formation and asexual embryogenesis through the regulation of key developmental genes such as Wuschel (Pasternak and Dudits). In addition to somatic embryogenesis, the epigenome also controls the juvenile-to-adult developmental transition notably by modulating the expression of regulatory genes. Indeed, in Arabidopsis plants, this transition is regulated by miR156/157 and its target-squamosa promoter binding protein-like gene (Xu et al.). Other epigenetic changes controlling the juvenile-to-adult developmental transition include DNA methylation, and histone modification (Xu et al.). Ultimately, these chemical changes lead to a remodeling of the chromatin. The SWI/SNF chromatin remodeling complexes play a central role in this biological process by controlling phytohormone biosynthesis, the establishment and maintenance of meristems, organ development, and floral transition (Ojolo et al.; Maury et al.). Supporting the central role of chromatin remodeling and histone modifications in controlling development of plant, Kang et al. studied the role of the chromatin-remodeling factor inositol auxotrophy 80 and the histone chaperones nap1-related protein 1 and 2 in modulating auxin fluxes and the activity of the inflorescence and root apical meristems. Another interesting study highlights the impact of the epigenome in controlling transcriptional initiation. The single-stranded DNA-binding protein whirly1 promoted the acetylation of H3K9 and repressed the trimethylation of H3K4 to enhance the recruitment of the RNA polymerase II on the wrky53 promoter (Huang et al.).

Epigenetic alterations also control the response of plants to environmental stresses including light perception and various abiotic stresses (e.g. salinity, drought, UV-B radiation, temperature, and heavy metal toxicity). As described by Lee et al. the circadian regulation of two proteins of the Sin3-histone deacetylase complex, encoded by SAP30 function-related 1 (*AFR1*) and *AFR2* genes, is critical for the proper regulation of Arabidopsis circadian rhythm. These two proteins directly bind to the circadaian clock associated 1 (*CCA1*) and pseudo-response regulator 9 (*PRR9*) promoters in order to locally deacetylate the histone H3 and negatively affect their expression. This is just a first level of the epigenetic regulation of the Arabidopsis circadian clock. Indeed, Hung et al. described a more complex transcriptional regulation of the circadian clock: the recruitment of the lysine-specific demethylase 1 (LSD1)-like 1/2 (LDL1/2) and histone deacetylase 6 (HDA6) proteins by circadian clock associated 1 (CCA1)/late elongated hypocotyl (LHY) is needed to repress the expression of timing of cab expression 1 (TOC1). Acting as a negative feedback regulatory loop, TOC1 also interacts with LDL1/2 and HDA6 proteins to repress the expression of *CCA1/LHY*. A broader picture of the role of the epigenome on the plant circadian clock is provided in the Du et al. review paper.

Environmental stresses also induce the formation of stress-responding agents such as nitric oxide. In soybean, Sun et al. revealed that the *de novo* deposition of trimethylated histone H3 lysine 27 residue in the promoter and coding sequence of plant genes is needed to repress their transcription in response to salt stress. Mechanistically, Ageeva-Kieferle et al. described in their review the role of nitric oxide as inhibitors of histone deacetylase through the S-nitrosation of selected cysteine residues. Nitric oxide also regulates the epigenome by controlling the expression of genes encoding DNA and histone methyltransferases and demethylases. Taken together, nitric oxide is a chemical agent controlling plant gene activity in response to environmental stresses notably by regulating the activity of various histone acetyltransferases, deacetylases, methyltransferases and demethylases, and DNA methyl transferases and demethylases.

## Concluding Remarks

This special topic clearly highlights the central role of the epigenome in the regulation of gene expression that influences many plant biological processes such as plant development and plant response to environmental stresses. A deeper analysis of the chromatin remodeling and transcription related mechanisms will be needed to better understand the epigenetic regulation of gene expression. Single cell -omic technologies such as single cell RNA-seq and ATAC-seq will enable further discoveries by capturing the transcriptome and epigenome for each cell composing a complex organ. While single cell RNA-seq was recently applied on Arabidopsis root protoplasts, there is a need to develop plant single cell ATAC-seq technology to gain a more complete picture of the plant cell epigenome.

## Author Contributions

All authors listed have made a substantial, direct and intellectual contribution to the work, and approved it for publication.

## Funding

The work was supported by Youth Innovation Promotion Association, Chinese Academy of Sciences (2017399), Guangdong Natural Science Foundation (2018A030313350), the Strategic Priority Research Program of the Chinese Academy of Sciences (XDA13020603), the National Science Centre, Poland UMO-2014/13/B/NZ2/01187, Natural Sciences and Engineering Research Council of Canada (06679), the Chinese University of Hong Kong direct grant for research (#4053383), the National Science Foundation (awards #1854326 and #1339194), the Spanish Ministry of Economy, Industry and Competitiveness-INIA-FEDER (RTA2017-00011-C03-01) and by the Nebraska Research Initiative core facility research grant.

## Conflict of Interest

The authors declare that the research was conducted in the absence of any commercial or financial relationships that could be construed as a potential conflict of interest.

